# Association between Inappropriate End-of-Life Cancer Care and Specialist Palliative Care: A Retrospective Observational Study in Two Acute Care Hospitals

**DOI:** 10.3390/cancers16040721

**Published:** 2024-02-08

**Authors:** Manon S. Boddaert, Heidi P. Fransen, Ellen J. M. de Nijs, Dagmar van Gerven, Leontine E. A. Spierings, Natasja J. H. Raijmakers, Yvette M. van der Linden

**Affiliations:** 1 Netherlands Comprehensive Cancer Organisation (IKNL), 3501 DB Utrecht, The Netherlands; 2 Center of Expertise in Palliative Care, Leiden University Medical Center, 2333 ZA Leiden, The Netherlands; 3 Netherlands Association for Palliative Care (PZNL), 3501 DB Utrecht, The Netherlands; 4 Department of Medical Oncology, Alrijne Hospital, 2353 GA Leiderdorp, The Netherlands

**Keywords:** oncology, clinical decisions, specialist palliative care, end-of-life care, inappropriate care

## Abstract

**Simple Summary:**

A substantial number of patients with cancer receive inappropriate end-of-life care. Improving the quality of end-of-life care is a priority for both patients and their families. Palliative care has been demonstrated to improve the quality of life of patients with a life-threatening illness or frailty. The aim of our retrospective study was to assess whether cancer patients who were provided with specialist palliative care more than a month before their death were less likely to receive inappropriate end-of-life care than patients who were not. We analysed the hospital administrative data of 2603 deceased patients with cancer and found that 690 patients (27%) received potentially inappropriate care in their last month of life. Specialist palliative care was provided to 359 patients (14%). The likelihood for them to receive inappropriate end-of-life care was 45% lower than for patients who were not provided with specialist palliative care.

**Abstract:**

A substantial number of patients with life-threatening illnesses like cancer receive inappropriate end-of-life care. Improving their quality of end-of-life care is a priority for patients and their families and for public health. To investigate the association between provision, timing, and initial setting of hospital-based specialist palliative care and potentially inappropriate end-of-life care for patients with cancer in two acute care hospitals in the Netherlands, we conducted a retrospective observational study using hospital administrative databases. All adults diagnosed with or treated for cancer in the year preceding their death in 2018 or 2019 were included. The main exposure was hospital-based specialist palliative care initiated >30 days before death. The outcome measures in the last 30 days of life were six quality indicators for inappropriate end-of-life care (≥2 ED-visits, ≥2 hospital admissions, >14 days hospitalization, ICU-admission, chemotherapy, hospital death). We identified 2603 deceased patients, of whom 14% (*n* = 359) received specialist palliative care >30 days before death (exposure group). Overall, 27% (*n* = 690) received potentially inappropriate end-of-life care: 19% in the exposure group, versus 28% in the non-exposure group (*p* < 0.001). The exposure group was 45% less likely to receive potentially inappropriate end-of-life care (AOR 0.55; 95% CI 0.41 to 0.73). Early (>90 days) and late (≤90 and >30 days) initiation of specialist palliative care, as well as outpatient and inpatient initiation, were all associated with less potentially inappropriate end-of-life care (AOR 0.49; 0.62; 0.32; 0.64, respectively). Thus, timely access to hospital-based specialist palliative care is associated with less potentially inappropriate end-of-life care for patients with cancer. The outpatient initiation of specialist palliative care seems to enhance this result.

## 1. Introduction

Over recent decades, concern has grown that when patients with life-threatening illnesses such as advanced cancer near the end of their life, life-prolonging medical treatments often prevail over comfort-oriented care [1,2]. Disease-directed treatments or interventions that are appropriate to prolong life or treat disease-related symptoms for patients in good clinical condition may evolve into inappropriate interventions at the end of life, as possible negative effects outweigh the expected benefits [3]. Aside from reducing quality of care and ultimately the patient’s quality of life [4,5], this potentially inappropriate end-of-life care also raises economic and ethical concerns, as healthcare resources are spent on interventions providing little benefit and even potential harm, rather than on care that would be more appropriate for a patient at that stage [6].

Palliative care improves the quality of life of patients with a life-threatening illness or frailty through prevention and relief of suffering by means of early identification, careful assessment and treatment of symptoms of a physical, psychosocial, and spiritual nature, and facilitation of complex decision-making and advance care planning [7,8]. Several randomised and matched controlled trials have demonstrated that integration of specialist palliative care (SPC) into oncology care leads to improved quality of life and more appropriate end-of-life care for patients with advanced cancer [9,10,11]. This is found especially when SPC is provided early and regularly [9,10,11], and when it is initiated in outpatient setting rather than in inpatient setting [12,13,14]. 

In the Netherlands, palliative care is provided in all care settings, mostly by health care professionals without formal palliative care training, so-called generalists in palliative care [15,16,17]. These generalists in palliative care provide basic management of physical and psychological symptoms, and have basic discussions about prognosis and goals of treatment [16]. For patients with cancer, generalist palliative care will be provided by their hospital-based oncologist as well as their general practitioner. To support these clinicians, professional standards and guidelines for palliative care are available, and every Dutch hospital providing cancer care is required to have a multidisciplinary SPC team available to provide additional support [18]. These teams may offer expertise in management of refractory pain, other complex physical and psychological symptoms, existential stress, conflict resolution regarding goals of treatment, and discussions concerning situations of near futility [16]. An SPC team should consist of at least two medical specialists and a nurse or nurse practitioner with specific expertise in palliative care [18]. Studies have shown that referrals to specialist palliative care in the Netherlands are triggered by the complexity of patients’ needs, regardless of cancer type or prognosis at diagnosis, and frequently do not occur until the last month of life [19,20,21]. Previous research showed that, on average, less than 1 percent of the total annual number of admitted patients in Dutch hospitals were referred to SPC teams, whereas a referral rate of 3–4% would seem more appropriate based on SPC utilisation data from the UK, Australia, and the USA [20,22,23,24,25]. A recent population-based observational study showed a higher percentage of potentially inappropriate end-of-life care for patients with cancer in the Netherlands compared to Canada (34% vs. 22%) [26,27]. Only 9% of all deceased patients with cancer in the Dutch study received SPC in the year preceding their death, compared to 29% in Canada and 47% in Belgium [28,29]. As it is known from controlled studies that patients with cancer or other life-limiting diseases who are provided with SPC have lower healthcare utilisation at the end of life [9,30,31,32], potential under-utilisation of SPC services may contribute to this high proportion of patients receiving potentially inappropriate end-of-life care in the Netherlands. In the Netherlands Quality Framework for Palliative Care, the surprise question “Would I be surprised if this patient died in the next 12 months?” has been proposed as practical instrument to identify patients with potential palliative care needs, when the answer to the question is “no” [33,34]. This specific tool was incorporated to trigger and improve early identification. Gaining a better understanding of SPC provision and its benefits may increase awareness for referral and contribute to improving quality end-of-life care. 

The aim of this study was to assess the association between hospital-based SPC provision, timing and initial setting, and potentially inappropriate end-of-life care in cancer patients in two acute care hospitals in The Netherlands. We hypothesised that the provision of hospital-based SPC is associated with less potentially inappropriate end-of-life care, and that early provision and initiation in the outpatient setting may have an enhancing effect.

## 2. Materials and Methods

### 2.1. Study Design

We conducted a multicentre retrospective observational study using hospital administrative data to evaluate healthcare utilisation at the end-of-life and specialist palliative care provision in the year prior to death in 2018 or 2019. 

### 2.2. Study Setting and Participants

The study was conducted in two acute care hospitals in the Netherlands: one university medical centre and one general hospital. Both hospitals have between 20,000–25,000 admissions per year, and in 2017 had an annual referral rate to their SPC team of 1.5%, which were both in the top 25% of SPC referral rates of Dutch hospitals [20].

All adult deceased patients who were registered in these hospitals at the time of their death in 2018 or 2019 were included, providing their electronic medical record showed an ICD-10 code indicating diagnosis or treatment for solid malignancies (i.e., ICD-10 codes C00–C43 and C45–C76) or metastases (C77–C80) in the year preceding death [35]. The latter group includes both unknown primary cancers and so-called malignancies of other secondary and unspecified sites. As treatment strategies and disease trajectories for patients with haematological malignancies tend to differ from patients with solid malignancies, these patients were excluded. In addition, patients with basal cell carcinoma of the skin were excluded, as this diagnosis normally does not progress to advanced cancer and these patients probably died through other non-cancer causes.

### 2.3. Data Source and Extraction

Data were derived from HiX^®^ (healthcare information exchange) electronic medical records stored in a single clinical data repository in each hospital. Data intelligence units in both participating hospitals built a research specific query for data extraction. In consideration of previous study results and the aforementioned ‘surprise question’ as a tool to trigger early identification [19,20,21], the query was built to extract data on provision, timing and intensity, and the initial setting of SPC over a period of one year preceding the date of death. 

Data collection on potentially inappropriate end-of-life care was restricted to the last 30 days of life. Collected data from both hospitals were deidentified before analysis.

### 2.4. Specialist Palliative Care Provision

In both participating hospitals, the SPC team consisted of specifically trained nurses and nurse practitioners providing inpatient and outpatient consultations, in co-management with specifically trained medical specialists or primary care physicians. All new patients were discussed in a weekly multidisciplinary team meeting. 

Provision of SPC was assessed by use of (1) specific national Diagnosis-Treatment Combination (DTC) codes required for the reimbursement of SPC in a hospital setting (Appendix A, Table A1), and (2) specific appointment codes administratively attached to each consultation provided by the SPC team (Appendix A, Table A2).

DTC codes for disease-directed treatments with palliative intent (e.g., palliative chemotherapy or palliative radiotherapy) were considered part of usual care by medical oncologists and radiation oncologists and were therefore not defined as specialist palliative care provision. Similarly, generalist palliative care provision by oncologists may be expected to be part of usual care as well.

### 2.5. Exposure Group

To compose the exposure group, we distinguished between specialist palliative care initiated >30 days before death, specialist palliative care initiated ≤30 days before death, and no palliative care at all. Patients for whom SPC was initiated >30 days before death were assigned to the exposure group.

### 2.6. Non Exposure Group

**Figure 1:**
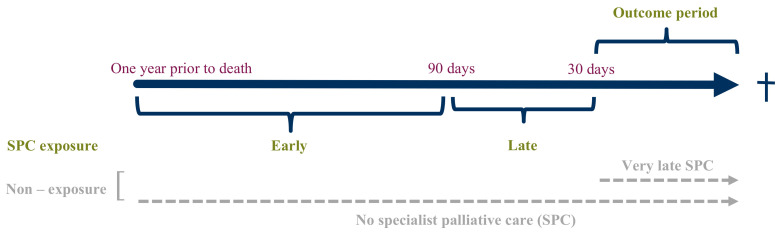
Specialist palliative care exposure and non-exposure group.

Patients were allocated to the non-exposure group when they had no registrations for SPC in the year before death or when SPC was initiated ≤30 days before death (very late SPC) (Figure 1). This was done to ensure the exposure (receiving SPC) was initiated before measuring the outcome (potentially inappropriate care in the last 30 days of life).

### 2.7. Timing of Specialist Palliative Care Provision

To assess the influence of timing on potentially inappropriate end-of-life care, we performed a subgroup analysis of early and late initiation of SPC in the exposure group compared to the non-exposure group. In accordance with previous studies, we defined early palliative care as initiated >90 days before death [12,13] and late palliative care as initiated ≤90 and >30 days before death. As mentioned, very late palliative care was separately defined as initiated ≤30 days before death and assigned to the non-exposure group (Figure 1).

To report the intensity of SPC provision in the exposure group, we also assessed the median number of SPC consultations per patient for each timeframe (i.e., number of SPC consultations >90 days, ≤90 and >30 days, and ≤30 days before death).

### 2.8. Initial Setting of Specialist Palliative Care Provision

To assess association between the initial setting of SPC provision and potentially inappropriate end-of-life care, we conducted a subgroup analysis for the inpatient and outpatient initiation of SPC in the exposure group compared to the non-exposure group. Subgroups were composed based on inpatient and outpatient appointment codes that were administratively linked to the initial consultation provided by the SPC team (Appendix A, Table A2).

### 2.9. Outcome Measures

To assess the quality of care in the last 30 days of life, we selected six population-based quality indicators measuring potentially inappropriate end-of-life care: provision of chemotherapy, frequency of emergency room visits (≥2) and hospital admissions (≥2), length of hospitalisation (>14 days), intensive care unit admissions (≥1), and hospital death. These indicators were based on previous studies regarding the development, validation, and benchmarking of these indicators [36,37,38,39]. Patients scoring any of these items were defined as receiving potentially inappropriate end-of-life care [12,26,37]. In addition, the mean composite score was calculated, representing the mean number of indicators per patient [26]. 

In preparation for analysis, databases from both hospitals were merged and adapted: (1) data from patients that had been registered in both hospitals were combined to avoid duplicates, (2) admission and discharge on the same day was considered as day care and therefore not counted as an admission, (3) for patients with missing admission data but a registered death in the hospital, the number of admissions was set at one, and (4) registered admissions that started >30 days before death and continued within the timeframe of the last 30 days were counted as admission. The subsequent number of hospitalisation days was calculated from day 30 before death to the date of discharge.

### 2.10. Statistical Analysis

We used descriptive statistics to assess potentially inappropriate end-of-life care and provision, timing and intensity, and the initial setting of SPC. To test for differences, we used the chi-squared test (categorical variables) and *t*-test (continuous variables). To assess the association between SPC provision, timing, and initial setting and receiving potentially inappropriate end-of-life care, a multivariable logistic regression was used. Adjusted odds ratios (AORs) and the corresponding 95% Cis are reported. To control for case-mix variations in our model, we adjusted for age, sex, and cancer diagnosis. A 2-tailed *p* value less than 0.05 was considered statistically significant. All analyses were conducted in SPSS (version 25.0.0.2.).

## 3. Results

In total, 2603 patients diagnosed with or treated for cancer in the year preceding their death in 2018 or 2019 were included. The mean age was 72 years (range 18–97), and most patients were male (56%). The three most prevalent cancer diagnoses were non-colorectal gastro-intestinal cancers (19%), lung cancer (14%), and cancers of the genito-urinary tract (12%) (Table 1).

### 3.1. Specialist Palliative Care Provision

In total, 792 patients (30%) received SPC in the last year of life. In 359 patients (14%) SPC was provided >30 days before death (exposure group), of which 6% was initiated early (>90 days before death) and 8% late (≤90 and >30 days before death). The remaining 433 patients (17%) were provided with very late SPC (≤30 days before death) and duly assigned to the non-exposure group (Table 1).

In the exposure group, deceased patients were younger and more often female. Gynaecologic cancer was more prevalent in the exposure group, 8% vs. 5% (*p* = 0.035), whereas genito-urinary tract cancer and breast cancer were more prevalent in the non-exposure group, 8% vs. 13% (*p* = 0.02) and 3% vs. 6% (*p* = 0.024), respectively (Table 1).

**Table 1:** Sociodemographic and clinical cohort characteristics.

Characteristic	Total	Exposure Group ^a^	Non-Exposure Group ^b^	*p*-Value
	N %	N %	N %	
*Overall no. of decedents*	2603 100	359 14	2244 86	
*Age*				
Mean (range)	72 (18–97)	67 (21–95)	73 (18–97)	**<0.001**
*Sex*				**<0.001**
Male	1461 56	169 47	1292 58	
Female	1142 44	190 53	952 42	
Prevalence cancer diagnoses *				
Non-colorectal GI cancers **	492 19	78 22	414 18	0.141
Lung cancer	359 14	55 15	304 14	0.366
Genito-urinary tract cancer	314 12	30 8	284 13	**0.020**
Colorectal cancer	236 9	37 10	199 9	0.378
Breast cancer	155 6	12 3	143 6	**0.024**
Gynaecologic cancer	136 5	27 8	109 5	**0.035**
Melanoma	106 4	13 4	93 4	0.641
Other cancers ***	565 22	65 18	500 22	0.075
Metastases ^$^	240 9	42 12	198 9	0.080
*Specialist palliative care*				
Early ^c^	165 6	165 46		
Late ^d^	194 8	194 54		
Very late ^e^	433 17		433 19	
None	1811 69		1811 81	

^a^ SPC initiated >30 days before death. ^b^ SPC initiated ≤30 days before death or not at all. ^c^ Initiated >90 days before death. ^d^ Initiated ≤90 days and >30 days before death. ^e^ Initiated ≤30 days before death. * Based on registered ICD-10 code in the last year of life. ** GI: gastro-intestinal. *** aggregated group of diagnoses: prevalence <3% per diagnosis. ^$^ Includes both unknown primary cancers and so-called malignancies of other secondary and unspecified sites.

### 3.2. Potentially Inappropriate End-of-Life Care

Of all 2603 patients, 690 (27%) experienced potentially inappropriate end-of-life care during the last 30 days of life, 19% in the exposure group (*n* = 359), and 28% patients in the non-exposure group (*n* = 2244), (*p* < 0.001). Table 2 lists the six quality indicators for potentially inappropriate end-of-life care. ICU-admission (1% vs. 7%, *p* < 0.001) and hospital death (6% vs. 18%, *p* < 0.001) occurred less often in the exposure group compared to the non-exposure group.

### 3.3. Timing of Specialist Palliative Care Provision

In the exposure group, SPC was initiated early (>90 days before death) in 46% of cases (Table 3). Patients receiving early SPC had a mean total of seven consultations before death, and patients receiving late SPC had five. No differences in the prevalence of potentially inappropriate end-of-life care or mean number of individual quality indicators per patient were found between patients receiving early or late SPC.

### 3.4. Initial Setting of Specialist Palliative Care Provision

In the exposure group, 26% of SPC provision was initiated in the outpatient setting (Table 4). Patients for whom palliative care was initiated in the inpatient setting more often received potentially inappropriate end-of-life care compared to those for whom it was initiated in the outpatient setting, respectively, 22% vs. 12%, (*p* = 0.037). On average, SPC was initiated 4.4 months before death in the outpatient group and 3.4 months in the inpatient group.

### 3.5. Association between Provision, Timing, and Setting of Specialist Palliative Care and Receiving Potentially Inappropriate End-of-Life Care

Adjusted for age, sex, and cancer diagnosis, patients receiving SPC more than 30 days before their death (exposure group) were 45% less likely to experience potentially inappropriate end-of-life care (adjusted OR 0.55; 95% CI 0.42 to 0.74) compared to patients who received no SPC or received SPC less than 30 days before their death (non-exposure group). 

**Figure 2:**
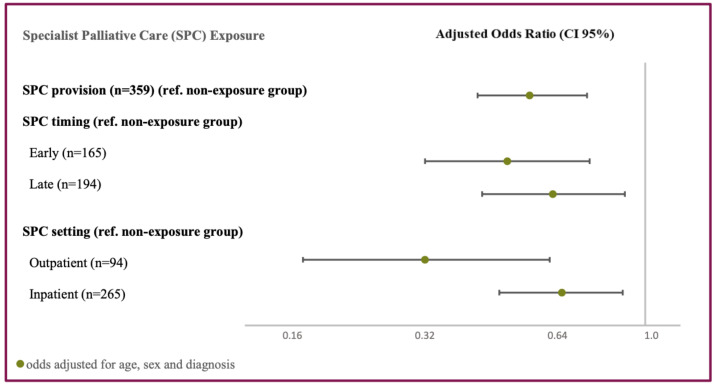
Association between specialist palliative care and potentially inappropriate end-of-life care.

Subgroup analysis of the exposure group showed similar odds for both early (>90 days) and late (>30 and ≤90 days) SPC initiation (AOR 0.49; 95% CI 0.32 to 0.75 and 0.62; 95% CI 0.43 to 0.90, respectively) as for inpatient initiation (AOR 0.65; 95% CI 0.47 to 0.89) compared to the non-exposure group (Figure 2). Patients for whom SPC was initiated in the outpatient setting appeared three times less likely to receive potentially inappropriate end-of-life care compared to the non-exposure group (AOR 0.32; 95% CI 0.17 to 0.61).

## 4. Discussion

This study showed that more than one fourth of deceased patients with cancer in two acute care hospitals in the Netherlands receive potentially inappropriate care in their last month of life. Of all deceased patients with cancer, nearly one third receives specialist palliative care, of which 14% received it prior to their last month of life. Patients who received SPC before their last month of life were nearly two times less likely to experience potentially inappropriate care in the last month of their lives compared to patients who receive no SPC or received it only in their last month of life. Our results suggest that the initiation of SPC provision in the outpatient setting might further enhance these odds, whereas most patients received SPC in the inpatient setting.

Remarkably, the highest proportion of decedents in our study population consisted of patients with non-colorectal gastro-intestinal cancer (Table 1). This may be a result of the tertiary-referral capacity of the university medical centre involved in this study. Moreover, our data concern prevalent cancer diagnoses in deceased patients, which may differ from prevalence at diagnosis due to progressiveness of diseases. As the subsequent prevalence of cancer diagnoses in our data are in line with overall prevalence in the Netherlands, we believe our data can still be considered generalisable for comparison to other hospitals [40].

Our results show an overall proportion of 31% of patients with advanced cancer received hospital-based SPC in the year prior to their death. In a previous population-based observational study, we assessed national data across care settings and found that, of all patients with cancer in The Netherlands who died in 2017, only 9% were provided with SPC in the year prior to their death, compared to 29% in Canada and 47% in Belgium [27,28,29]. In view of complex reimbursement regulations for hospital-based SPC teams and the observed low referral rates to these teams in a previous hospital survey [20], under-registration and underutilisation of specialist palliative care were hypothesised to account for the low percentage of SPC provision and the high proportion (34%) of patients receiving potentially inappropriate end-of-life care in this study.

Our current findings mainly support the hypothesis of general underutilisation of SPC in our previous study, as a higher degree of timely hospital-based SPC utilisation (14%) is associated with a lower proportion of patients receiving potentially inappropriate end-of-life care (27%).

In a systematic literature review and meta-analysis addressing the association between palliative care and patient and caregiver outcomes, palliative care was consistently associated with lower healthcare utilisation, as well as with improved patient and caregiver satisfaction [41]. In line with our findings, several studies have recently used administrative databases and demonstrated an association between palliative care and healthcare utilisation at the end of life, both for patients with cancer as for patients with non-cancer diseases [27,29,30,32,42,43,44]. 

Addressing the effect of the timing of palliative care provision on quality end-of-life indicators, a recent study indicated patients provided with either generalist or specialist palliative care more than one month before their death were less likely to be admitted or die in hospital [30]. These findings are in line with our current hospital-based study, as well as our previous study across care settings [27]. Contrary to other studies, our study did not show a significant improvement in the quality of end-of-life care through the earlier (>3 months) initiation of SPC [12,13,30,44]. This may be attributed to the relatively small number of patients in the early–late SPC analysis. However, these results are consistent with the findings in our previous nation-wide population-based study [27]. 

While using similar definitions for early and late palliative care to previous studies, these studies did not exclude palliative care provided during the outcome period (i.e., last 30 days before death) from the late palliative care group [12,13,44]. This may have reflected positively on the outcomes for potentially inappropriate end-of-life care in patients with early SPC, as patients provided with SPC in the last 30 days before death may have more unstable conditions and would thus be more likely to receive acute hospital care at the end of life. Results from multiple randomised trials have also emphasised the positive effect of early SPC. However, in these studies, SPC was not only initiated shortly after diagnosis of advanced cancer; it was also initiated in the outpatient setting [9,10,45,46].

When looking at setting, in our study, 26% of patients provided with SPC were initiated in the outpatient clinic. Adjusted for age, sex, and diagnosis, these patients were 68% less likely to receive potentially inappropriate end-of-life care compared to the non-exposure group. Although this analysis comprised a relatively small population, a previous study among 366 deceased patients assessing both early and outpatient SPC found that only outpatient SPC provision was independently associated with less potentially inappropriate end-of-life care [12]. More recently, similar results were reported in a study of 327 patients, where specifically SPC exposure in the outpatient setting was linked to shorter hospital length of stay and lower ICU admissions [14]. 

Thus, our results appear to corroborate the importance for outpatient SPC involvement.

Deciding when to forego medical treatments as the disease progresses and death comes near is often a difficult and complex decision. A systematic review on the extent of non-beneficial treatments in acute hospitals at the end of life confirmed widespread occurrence [47]. And despite increased emphasis to reduce inappropriate end-of-life care in the past decade, a recent large population-based study among more than 146,000 persons aged 66 years and older with advanced cancer demonstrated such care remains very common [48]. Several randomised and matched controlled trials have demonstrated that the integration of specialist palliative care into oncology care leads to improved quality of life and more appropriate end-of-life care for patients with advanced cancer [9,10,11]. For that reason, professional organisations for oncology recommend earlier and routine co-management of patients by oncologists and palliative care specialists [49,50]. We believe our results corroborate the conclusions from a systematic review of these randomised trials, indicating that interdisciplinary care provided in a concurrent two-track approach by both oncologists and palliative care specialists can improve appropriate end-of-life care based on patients’ preferences [41].

### Strengths and Limitations

The use of electronic medical records covering all patients registered in the participating hospitals enabled us to assess the quality of end-of-life care for a cohort of patients. In addition, it minimised selection bias and rendered our findings generalisable for comparison to other hospitals treating patients with cancer. Designing a feasible data query tailored to answer the research aims required careful coding of the indicators and multiple checks for accuracy. To enable this process, our multidisciplinary research team comprised data scientists, healthcare professionals, an epidemiologist, and a reimbursement administrator. However, some limitations need to be addressed. Firstly, population-based quality indicators were used on an aggregated level and cannot be used as indicators of inappropriate care for individual patients. A patient’s goals of care may well be known, personal preferences may differ, and clinical factors may justify acute care interventions. Therefore, we strictly adhered to the term ‘potentially’ inappropriate end-of-life care. Secondly, rather than prospectively collecting data in a randomised design to answer our research questions, we extracted our data retrospectively from administrative databases not primarily designed for the purpose of quality assessment. Thus, a general limitation resulted from a lack of clinical information about the complexity of needs and content of care provided, the awareness of healthcare professionals about their patients being in their last months of life, the willingness of patients to accept specialist palliative care, and whether patients ultimately died because of their cancer diagnosis or of other causes. Subsequently, we could not control for disease-related confounders such as performance status, illness severity, or prior cancer treatment, and confounding by indication may therefore be present [51]. Although we controlled for age, sex, and diagnosis in our analyses, we did not take ‘time since diagnosis’ into account as a potential confounding factor. A shorter time since diagnosis may focus physicians and patients more on disease-directed treatments than on comfort-oriented care. In support of our results, a large study with a similar design to our research did control for time since diagnosis in patients with gastro-intestinal cancer, and similarly found significantly less healthcare utilization, including ICU admissions and hospital deaths, for patients receiving palliative care [52]. Thirdly, we collected our data in just two acute care hospitals, resulting in a limited number of patients per individual indicator in the exposure group and in our sub analyses. In our overall results, the SPC intervention significantly reduced ICU admission and hospital death, but failed to prevent emergency admissions, hospital admissions, >14 days of hospitalization, and chemotherapy during the last month of life. We believe these results may be attributed to the limited number of patients per individual indicator in the exposure group, as similar but larger studies within our research group showed significant reduction for more or all individual indicators with similar SPC interventions, depending on the size of the population [27,53,54].

Finally, recent population-based studies have indicated that patients who received inpatient palliative care within six months prior to their death were more likely to access community palliative care after discharge than those who received no inpatient palliative care [55,56]. The receipt of community palliative care after hospital discharge has been shown to decrease readmissions and health care utilisation [57,58]. Therefore, outside the scope of this study, continuity of palliative care in the community may have added to our results.

## 5. Conclusions

This study shows that referrals to specialist palliative care for patients with cancer mostly occur late in the disease trajectory and in the inpatient setting. Initiation of specialist palliative care prior to the last month of life is associated with less potentially inappropriate end-of-life care in the last month of life. Initiation in an outpatient setting may further enhance these odds. These results imply a need to improve access to specialist palliative care prior to the last month of life. Future prospective studies should examine the differences in the disease trajectory (e.g., time since diagnosis) and underlying characteristics of exposure and non-exposure of specialist palliative care recipients to improve insight into the most effective model to provide SPC. This would allow for the earlier identification of patients who may benefit from timely palliative care in an interdisciplinary generalist–specialist palliative care model where referrals are based on the complexity of palliative care needs.

## Figures and Tables

**Table 2:** Quality indicators of potentially inappropriate end-of-life care ^a^.

Characteristics	Total	Exposure Group	Non-Exposure Group	*p*-Value
	N %	N %	N %	
*Overall no. of decedents*	2603 100	359 14	2244 86	
*Inappropriate EoL* ^b^ *care* ^c^				
Yes	690 27	68 19	622 28	**<0.001**
No	1913 73	291 81	1622 72	
*Indicators*				
≥2 emergency room visits	115 4	15 4	100 5	0.812
≥2 hospital admissions	244 9	32 9	212 9	0.747
>14 days of hospitalisation	200 8	22 7	178 8	0.233
Chemotherapy	112 4	16 5	96 4	0.877
ICU admission	157 6	4 1	153 7	**<0.001**
Hospital death	430 17	21 6	409 18	**<0.001**
*Mean composite score (SD)* ^d^	1.8 (0.96)	1.6 (0.93)	1.8 (0.96)	0.064
*First initiation of SPC* ^e^ *(mean)* ^f^	1.8	3.7	0.3 ^g^	

^a^ In the last 30 days before death. ^b^ EoL; end-of-life. ^c^ Qualification is rendered positive when 1 out of 6 indicators are scored. ^d^ Total amount of 6 indicators divided by number of patients receiving potentially inappropriate end-of-life care. ^e^ SPC; specialist palliative care. ^f^ In months before death. ^g^ Based on 433 patients with very late SPC (initiated ≤30 days before death (Table 1)).

**Table 3:** Quality indicators for potentially inappropriate end-of-life care ^a^ in the exposure group (*n* = 359) in relation to timing of specialist palliative care.

Characteristics	Early SPC ^b^	Late SPC ^c^	*p*-Value
	N %	N %	
*Overall no. of decedents*	165 46	194 54	
*Inappropriate EoL* ^d^ *care* ^e^			
Yes	29 18	39 20	0.542
No	136 82	155 80	
*First initiation of SPC (mean)* ^f^	5.9	1.8	
*Number of SPC consultations >* 3 *months* ^g^	2 (1–3)	-	
*Number of SPC consultations* 3–1 *months* ^g^	2 (1–4)	2 (1–3)	
*Number of SPC consultations <* 1 *month* ^g^	3 (2–4)	3 (1–4.5)	

^a^ In the last 30 days before death. ^b^ Early specialist palliative care initiated >90 days before death. ^c^ Late specialist palliative care initiated ≤90 days and >30 days before death. ^d^ EoL; end of life. ^e^ Qualification is rendered positive when 1 out of 6 indicators are scored. ^f^ In months before death. ^g^ Median and interquartile range.

**Table 4:** Quality indicators for potentially inappropriate end-of-life care ^a^ in the exposure group (n= 359) in relation to initial setting of palliative care.

Characteristic	Outpatient	Inpatient	*p*-Value
	N %	N %	
*Overall no. of decedents*	94 26	265 74	
*Inappropriate EoL* ^b^ *care* ^c^			
Yes	11 12	57 22	**0.037**
No	83 88	208 78	
*First initiation of SPC* ^d^ *(mean)* ^e^	4.4	3.4	

^a^ In the last 30 days before death. ^b^ EoL; end of life. ^c^ Qualification is rendered positive when 1 out of 6 indicators are scored. ^d^ SPC; specialist palliative care. ^e^ In months before death.

## Data Availability

The manuscript, together with Appendix A, provide the study’s minimal data set, and will enable others to inform their own process of collecting and analysing the administrative data of health care utilisation and palliative care provision. The datasets generated and analysed during the current study are held securely by Leiden University Medical Center, Center of Expertise in Palliative Care, and are not publicly available due to confidentiality, but are available from the corresponding author on reasonable request. The lead authors (the manuscript’s guarantors) affirm that the manuscript is an honest, accurate, and transparent account of the study being reported; that no important aspects of the study have been omitted; and that any discrepancies from the study as originally planned have been explained.

## Appendix A

**Table A1:** Diagnosis–Treatment Combination reimbursement codes for specialist palliative care provision.

Reimbursement Code Specification	Code	Description	Billable for/by
*Diagnosis*	3130050	Palliative care	Internal medicine
*Diagnosis*	3309950	Palliative care	Neurology
*Diagnosis*	3890990	Palliative care	Anaesthesiology
*Diagnosis*	3229950	Palliative care	Pulmonary medicine
*Diagnosis*	3350352	Palliative care	Geriatric medicine
*Consultation activity*	190067	SPCT * consultation	Physician/NP *
*Consultation activity*	190006	SPCT meeting	Physician/NP
*Consultation product*	990040009	1–2 consultation(s)	Physician/NP
*Consultation product*	990040007	>2 consultations	Physician/NP
*Consultation product*	990040004	>1, with diagnostics	Physician/NP
*Consultation product*	990040006	>1, with intervention	Physician/NP
*Consultation product*	990040005	Day-care with intervention	Physician/NP
*Consultation product*	990040003	Admission	Physician/NP

* SPCT: Specialist palliative care team. * NP: Nurse practitioner.

**Table A2:** SPCT *: appointment codes for specialist palliative care provision per hospital.

Setting	Code	Description	Billable for/by
*Inpatient*	KEC	Initial consultation	SPCT General Hospital
*Inpatient*	KVC	Follow-up consultation	SPCT General Hospital
*Outpatient*	PEC	Initial consultation	SPCT General Hospital
*Outpatient*	PVC	Follow-up consultation	SPCT General Hospital
*Interdisciplinary*	ICC	Consultation between peers	SPCT General Hospital
*Inpatient*	KNP	Initial consultation physician	SPCT University Hospital
*Inpatient*	KNPVC/VS	Initial consultation nurse/NP *	SPCT University Hospital
*Inpatient*	KCO	Follow-up consultation physician	SPCT University Hospital
*Inpatient*	KCOVC/VS	Follow-up consultation nurse/NP	SPCT University Hospital
*Outpatient*	NP	Initial consultation physician	SPCT University Hospital
*Outpatient*	NPVC/VS	Initial consultation nurse/NP	SPCT University Hospital
*Outpatient*	CO	Follow-up consultation physician	SPCT University Hospital
*Outpatient*	COVC/VS	Follow-up consultation nurse/NP	SPCT University Hospital
*E-consult*	TP	Phone/E-consultation physician	SPCT University Hospital
*E-consult*	TPVC/VS	Phone/E-consultation nurse/NP	SPCT University Hospital
*Interdisciplinary*	ICC	Consultation between peers	SPCT University Hospital

* SPCT: Specialist palliative care team. * NP: Nurse practitioner.
